# From the Outgoing Editor-in-Chief of *Innovation in Aging*

**DOI:** 10.1093/geroni/igae103

**Published:** 2024-12-11

**Authors:** Steven M Albert

**Affiliations:** School of Public Health, University of Pittsburgh, Pittsburgh, Pennsylvania, USA

In 2017, the Gerontological Society of America (GSA) launched *Innovation in Aging* (*IA*), its first open-access online-only journal. *IA* was designed for “competitive papers that cross disciplines,” “high-quality papers not accepted in other GSA journals but worthy of publication,” and “papers with translational significance” (Sands, 2020). In its first 4 years, *IA* published 162 research papers. In its second, from 2021 when I began my role as Editor-in-Chief, *IA* published 319 (through October 2024). New submissions have more than tripled, from about 200 in 2017 to almost 700 by the end of this year. *IA* continues to publish 1–2 special issues annually and now publishes 12 issues a year, up from 4 in its first 4 years. *IA*’s 2023 Impact Factor is 4.9 (the 5-year impact factor of 6.4), ranking second in the Gerontology category.

*IA*’s special issues show the breadth of the journal. These cover a wide terrain: Aging in Sub-Saharan Africa (2024), Translational Research on Pain and Pain Management in Later Life (2023), Nursing Science Interventions in Aging (2022), Translational Research on the Future of U.S. Nursing Home Care (2022). All told, from 2020 to 2024, *IA* published 24 invited commentaries and editorials, many focusing on ways research in the special issues push the field to address new and neglected topics. A special issue covering opioid use among older adults is in the works for 2025.

Similarly, the most highly cited papers appearing in *IA* cover topics across the spectrum of aging science, again showing the versatility of the journal. Some of the most highly cited papers in the past 2 years include:

- “Using a Nature-Based Virtual Reality Environment for Improving Mood States and Cognitive Engagement in Older Adults: A Mixed-Method Feasibility Study” by Kalantari et al. (2022);
- “Putting the Nursing and Home in Nursing Homes” by Grabowski (2022); and
- “Family Caregivers in Rural Appalachia Caring for Older Relatives With Dementia: Predictors of Service Use” by Savla et al. (2021).

*IA* seeks innovation in publishing practice as well. Innovations in the last 4 years include the use of graphical abstracts, new keyword search functionality, policies on equity and inclusion, increased diversity among editorial board members and associate editors, and commitment to transparent policies on data access, use of artificial intelligence, and publishing ethics. Efficiency in the journal remains high, with a fast turnaround for decisions. Editors and reviewers take extra care to steward manuscripts to enhance scientific value. Authors mention how helpful comments are even for manuscripts rejected in the course of peer review.

None of this would be possible without a dedicated staff (especially, our Managing Editor, Karen Jung), committed Associate Editors and Editorial Board Members, and support from GSA and Oxford University Press. As I wrote earlier, “As an editor, I have a new appreciation for the sophisticated machinery required to publish timely, high-quality research” (Albert, 2021).

What can we learn from this experience that might inform the next 4 years, as we switch to new editorial leadership?

## Defining Translational Gerontology

*“IA* seeks research that draws on multiple disciplines to solve problems that stand in the way of optimal aging” (Albert, 2020). Our Instructions to Authors page stresses this point. In a required translational significance statement, authors are asked to specify implications for translation: “how the research will improve individual, organizational, societal, or environmental conditions associated with aging and the life course.” The goal is research that can help change some components of aging, “enhancing our ability to address a challenge posed by aging bodies, minds, relationships, or societies” (Albert, 2021).

Through the review of 2,000+ submissions as Editor-In-Chief over the past 4 years, I have identified a few touchstones for translation in gerontology that can help *IA* continue achieving its goals for publication. I present these as a guide that will no doubt continue to evolve.

**Figure 1.**
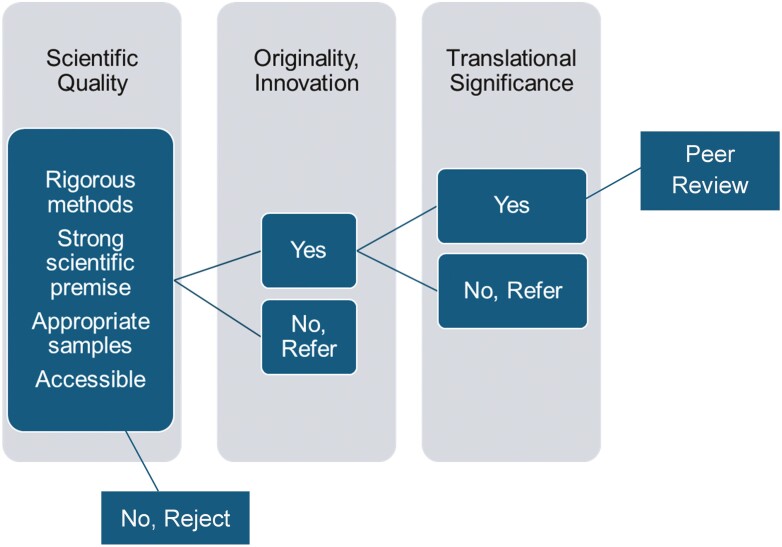
Triage criteria.

Figure 1, “Triage Criteria,” shows how *IA* differs from many journals in identifying research worthy of publication. Where reviews for many journals would stop at scientific quality, *IA* in addition insists on originality and translational significance. It is possible for research that meets the criteria for high scientific quality not to go out for peer review if it does not represent new and original research. Simply replicating a finding in a new setting or with a different subpopulation fails to meet the innovation-originality criterion. High-quality research that is not accessible, that is, that applies complex analytic approaches to an old research question without producing new insights, will also fail this test. Similarly, research too narrow in its approach to a question may fall short of originality.

Research that meets the originality criterion can still fall short in translational significance. Most typically, this involves high-quality science and an innovative approach that is unfortunately not guided by a clear clinical or policy issue. Even if carried out successfully, this research is unlikely to move the field in some direction. In this approach, *IA* follows the National Institutes of Health reviewer guidelines for “overall impact.” Papers that are good in scientific quality but fall short of originality or translational significance are returned to authors with recommendations for other more appropriate journals.

**Figure 2.**
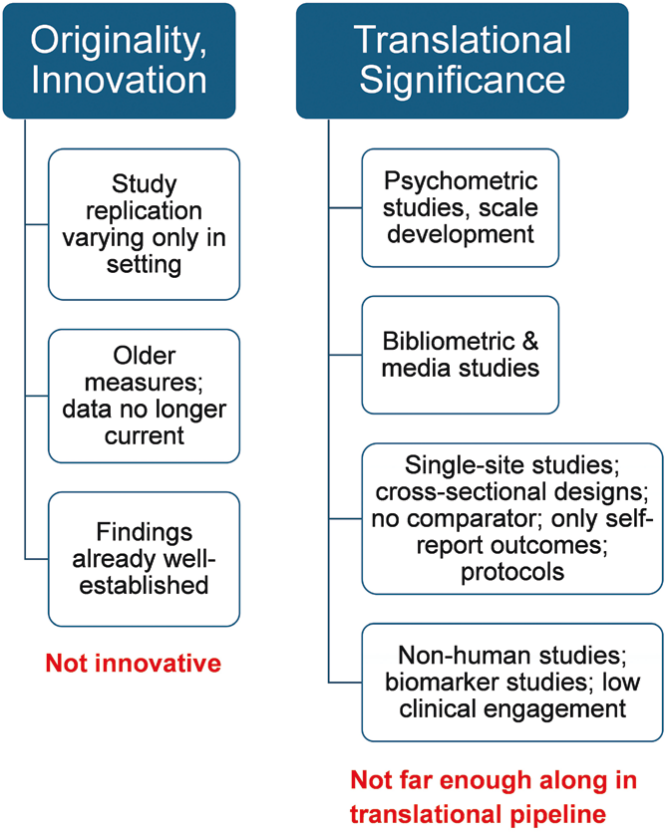
Features of research that lower originality and translational significance.

Figure 2 presents features of research that lower originality and translational significance. Many types of research do not meet the translational significance criterion because they are not far enough along in the translational pipeline. This is certainly true of protocol papers but extends as well to nonhuman studies or studies without patient-centered outcomes, such as studies simply assessing biomarkers or establishing typologies. Psychometric studies for scale development and bibliometric or media studies also fall short on translational significance. Other studies still early in the translational pipeline are valuable but need further maturation or development. These include studies only focusing on process measures rather than outcomes, or studies reporting community partnership or research registry efforts without showing effects on outcomes. Translational significance will be lower in studies relying on cross-sectional analyses, single-site recruitment, convenience samples, exclusively self-report outcomes, and pretest–posttest measures without a control condition or reasonable comparator. While iron-clad rules are not available for defining translational significance, these principles suggest guidelines for determining which manuscripts go out for peer review in a journal focused on translation.

## What Makes a Strong Contribution to *Innovation in Aging*?

Based on 24 years of editor-in-chief service, Rivera (2024) identified key elements of a strong research paper. He notes, “without doubt, the most important criterion in assessing a manuscript is whether or not the research question investigated is important in terms of improving health, health care, or public policy that affects health.” Substitute “aging” for “health” and we have excellent guidance to authors submitting to *IA*. A clear and significant research question is the start of all research and the key element in a successful route to publication.

Rivera goes on to make an important distinction in assessing a research question: “Well-done clinical trials that show no effect of an intervention can be very important by changing our care of patients or the policies that affect the population.” Built into a clinical trial, even one showing no effect, is a clear research question and hypothesis. By contrast, reporting results from large observational studies, with a myriad of measures and methods, does not guarantee that investigators have posed a worthwhile question. These studies often simply investigate a small gap in the literature (e.g., effect on a subpopulation, effect using an alternative measurement strategy, effect over a longer time period) rather than grapple with a clear, important research question. They offer only a small increase in knowledge and will face a harder time at *IA*.

## Parting Words

Orson Wells’s Citizen Kane rejected a good part of his wealthy inheritance, including lumber mills, steel plants, real estate, and factories, in favor of a newspaper. He remarked, “I think it might be fun to run a newspaper.” Serving as editor-in-chief of a major research journal is as close to running a newspaper as scientists get (though that may be changing in the new world of social media). It is a wonderful opportunity to see international scholarship, advances in science, and cross-disciplinary collegiality at close quarters. You cannot get too much closer to the center of the community of science. What a privilege and what a responsibility. Part of that responsibility also includes holding the line on publishing ethics, with the difficult but critical task of calling out duplicative (“salami”) publishing, plagiarism, and inappropriate authorship. These challenges are the exception but also part of the editor-in-chief’s remit.

As I leave the editor-in-chief position and welcome Michelle Putnam as the next editor (and say goodbye to my Deputy Editor-in-Chief, Jenny Stanley), I offer the following final thoughts.

To our authors: We appreciate your patience. Associate editors, reviewers, and journal staff process manuscripts as efficiently and as fairly as possible. Some papers require outreach to a dozen reviewers to get two and an inordinate amount of time for an appropriate review.

To our colleagues: Step up when asked to review. Again, getting two reviewers often requires outreach to more than a handful. It is critical that all of us participate in the peer review process. The same applies to invitations to join the editorial board as an editorial board member or an associate editor.

To our readers: Engage with the science. Each published paper represents hundreds, if not thousands, of hours of effort if we include data collection, the path to funding, and the full lifecycle of research. Pose a strong research question, answer it well, and submit to *IA*.

To our editorial staff and parent GSA: Thanks for your support and the excellent job you always do.
